# Pathogens Isolated and Their Association With the Long-Term Outcome in Patients With Acute Exacerbation of Chronic Obstructive Pulmonary Disease

**DOI:** 10.7759/cureus.26174

**Published:** 2022-06-21

**Authors:** C Mohan Rao, Kinshuk Sarbhai, Saswat Subhankar, Amrut Mohapatra, Nipa Singh, Prem S Panda, Shubhransu Patro, Sanghamitra Pati

**Affiliations:** 1 Pulmonary Medicine, Kalinga Institute of Medical Sciences, Bhubaneswar, IND; 2 Microbiology, Kalinga Institute of Medical Sciences, Bhubaneswar, IND; 3 Community Medicine, Kalinga Institute of Medical Sciences, Bhubaneswar, IND; 4 General Medicine, Kalinga Institute of Medical Sciences, Bhubaneswar, IND; 5 Epidemiology and Public Health, Regional Medical Research Centre, Bhubaneswar, IND

**Keywords:** long-term outcome, microbes, infections, acute exacerbations, copd

## Abstract

Background: Most of the acute exacerbations of chronic obstructive pulmonary disease (COPD) are due to infections, mostly due to bacteria and viruses. There is a need to study the outcome of microbe-induced airway inflammation.

Materials and methods: It is an observational follow-up study from the pulmonary medicine department of Kalinga Institute of Medical Sciences with the participation of the Regional Medical Research^ ^Center, Bhubaneswar, from October 2018 to February 2022. Patients who were admitted with acute exacerbation of COPD and treated as per GOLD (Global Initiative for Chronic Obstructive Lung Disease) 2021 guidelines were included in the study. Those patients in the severe category, who had clinically recovered, had undergone pulmonary physiotherapy, were on prescribed medications and home oxygen therapy after discharge, were followed up every three months by telephone calls. Any exacerbation, clinical stability, or mortality information was recorded.

Results: Out of 197 cases, the majority were elderly, males, smokers, and belonged to urban areas; in total, 102 (51.8%) microbes were isolated as etiological agents of infective exacerbation in which 19.79% were viruses and 23.35% were bacteria, while coinfection was found in 8.62% cases. Among the viruses, rhinovirus, influenza virus, and respiratory syncytial virus were the major isolates. Among the bacteria, mostly gram-negative organisms such as *Acinetobacter baumannii*, *Klebsiella pneumoniae*, and* Pseudomonas aeruginosa* were isolated. Readmission was more among patients with coinfection.

Conclusion: Acute exacerbation of COPD was mostly seen in males in the age group of 61-80 years. Rhinovirus and influenza A virus were the two most common viral isolates, and among the bacterial isolates, *Acinetobacter baumannii* and *Klebsiella pneumoniae *were predominantly detected. Poor clinical outcomes were noticed more among the coinfection group.

## Introduction

Worldwide, COPD is one of the major causes of illness and the sixth highest cause of death. According to research on the Global Burden of Diseases in 2017, it contributed to 50% of all chronic respiratory diseases. It is currently the third leading cause of death worldwide, accounting for nearly 3.23 million deaths, with nearly 80% of deaths occurring in the middle- and low-income countries, and is expected to rise from the 12th leading cause of disability-adjusted life-years (DALYs) in 1990 to the fifth leading cause in 2020 [1,2].

Acute exacerbations of COPD are significant events in the course of illness because they have a negative influence on health status, hospitalization rate, and disease progression. It is believed that respiratory infections are an important risk factor for COPD exacerbations, with viruses accounting for 22%-64% [3]. The increased exposure to viruses in winter has been correlated to an increase in the frequency of exacerbations in winter in some areas of the world [4]. Co-infections have also been linked to an increase in the severity of COPD exacerbations. The simultaneous discovery of bacteria and viruses in patients with acute exacerbation of COPD is responsible for the worsening lung function, prolonged hospital stay, and risk of recurrence of a similar event [5,6].

This study analyses the prevalence and pattern of viral and bacterial infections in patients presenting with acute exacerbation of COPD, correlates the type of infection with the severity of exacerbation among the patients, and finds out the long-term outcome of the severe follow-up cases after discharge in terms of readmission, clinical stability, or death.

## Materials and methods

The study was conducted from October 2018 to February 2022 among the patients admitted to critical care, Respiratory and General Medicine unit of Kalinga Institute of Medical Sciences, Bhubaneswar, in collaboration with Regional Medical Research Centre (ICMR), Bhubaneswar.

The sample size was calculated by using the formula: 

n = Z^2^ P(1−P)/d^2^

where n is the sample size; Z is the statistic corresponding to a 95% level of confidence, which is equal to 1.96; P is the expected prevalence (proportion of COPD patients with infectious etiology = 78.3% in a study conducted by Jahan et al.) [7]; d is the absolute precision (it has been taken as 6%). The sample size was found to be 179; adding a 10% non-response rate, the final sample size was 179 + 18 = 197.

Admitted cases underwent clinical assessment and other routine investigations. Empirical treatment was given as per standard treatment guidelines. The nasopharyngeal swab was taken and transported in a viral transport medium within 24 hours to the Regional Medical Research Centre (RMRC) for the detection of respiratory viruses. Samples were tested by real-time reverse transcription-polymerase chain reaction (RT-PCR). The test was done using recommended commercial kit (FTD, UK) following the manufacturer’s instructions on Applied Biosystems-7500 (ABI-7500) equipment (ABI, USA). After thorough rinsing of the oral cavity, respiratory secretions were sent in a sterile container to our institute laboratory for bacterial culture and sensitivity study by VITEK 2 compact instrument (bioMérieux, France).

Apart from the procedural guidelines, depending on the severity of the cases, patients were treated with microbe-targeted antibiotics, oxygen support, either parenteral or oral, nebulized corticosteroid, and bronchodilator and were classified as mild, moderate, and severe as per the GOLD guidelines. The severe cases underwent pulmonary physiotherapy (diaphragm strengthening, pursed-lip breathing, lower limb muscle training, and chest percussion) session one week after clinical stability.

The patients were contacted over telephonic/telemedicine services every three months (due to the COVID pandemic, physical follow-up was not done) to ensure that they were continuing to perform the exercises at home and consuming medications, and any clarifications sought were addressed. Outcome data were collected with respect to clinical stability, worsening of clinical symptoms requiring admission, or mortality at the end of one year of follow-up.

This is an observational follow-up study conducted in the pulmonary medicine department of the Kalinga Institute of Medical Sciences. Ethical clearance was obtained from Institutional Ethics Committee (vide letter no.: KIIT/KIMS/113). All patients (including those on ventilation) with acute exacerbation of COPD (based on acute onset of cough, increased sputum with or without purulence, and breathing difficulty) admitted to the pulmonary medicine department were included in the study. Patients with pulmonary tuberculosis (TB), bronchiectasis, bronchial asthma, pneumonia, and acute lung injury (based on history and evaluation) and patients unwilling to give consent were excluded from the study.

Statistical analysis

Descriptive statistics were done after the collection of data. Frequency distributions of categorical variables (occupation, gender, place of residence, smoking status, type of pathogens found, clinical features, comorbidities, and follow-up data) were calculated. For continuous data (age, total leukocyte count [TLC], and duration of hospital stays), mean and standard deviations were calculated. These were presented in tables using SPSS version 20.0 (IBM Corp., Armonk, NY) and Microsoft Excel 2007 (Microsoft Corporation, New Mexico, USA).

Type of infection, isolated organisms, and clinical outcomes after one year were identified. Chi-square and p-values were calculated to measure the associations between the type of infection and isolated organisms, type of infection, and readmission after one year.

## Results

A total of 197 subjects were included in the study, out of which 138 (70.06%) were males and 59 (29.94%) were females. The maximum number of subjects (130 [65.9%]) were within the age group of 61-80 years. The total number of patients more than 80 years of age was 25 (12.69%). The mean age of the patients was 69.24 ± 11.08 years (Table 1).

**Table 1:** Age and sex distribution of the study subjects (N = 197)

Age group (years)	Male	Female	Total
40-60	25	17	42
61-80	92	38	130
>80	21	4	25
Total	138	59	197

The total number of patients who had a smoking history was 126 (63.95%). Most of the study subjects were farmers (37.06%), and the least belonged to the category of laborer (2.54%). Out of the total subjects, only 83 (42.13%) patients were from rural areas (Table 2).

**Table 2:** Distribution of study subjects according to different variables (N = 197)

Variables	Frequency	Percentage (%)
Smoking history		
Smoker	126	63.96
Non-smoker	71	36.04
Occupation		
Teacher	16	8.12
Businessmen	21	10.66
Laborer	5	2.54
Farmer	73	37.06
Housewife	47	23.86
Unemployed	35	17.77
Area of residence		
Urban	114	57.87
Rural	83	42.13

Out of 197 patients,102 (51.78%) had been isolated with bacteria or viruses, or both. Isolated viral infection was seen in 39 (19.79%) cases, while 46 (23.35%) had only bacterial exacerbations. In another 17 (8.62%) cases, both bacteria and viruses were detected. No etiology for exacerbation could be detected in 95 (48.2%) cases (Table 3).

**Table 3:** Distribution of pathogens found in the subjects (N = 197)

Infection detected	No. of cases	Percentage (%)
Virus only	39	19.79
Bacteria only	46	23.35
Coinfection with both	17	8.62
No pathogen found	95	48.24
Total no. of patients	197	100

Out of 56 cases, in three cases of viral exacerbations, more than one virus (i.e., two) was detected, and in one case of viral exacerbation, more than one virus (i.e., three) was detected. A total of 62 viruses were isolated. Rhinovirus and Flu-A (H3N2) were isolated most frequently (30.35% and 25%, respectively) followed by respiratory syncytial virus (RSV) and parainfluenza virus 3 (PIV-3) (10.71% each; Table 4).

**Table 4:** Distribution of patients according to the type of virus isolated Note: Total may exceed the total number of patients affected with viral infection because of the isolation of multiple viruses. Flu-A: Influenza A virus; Flu-B: Influenza B virus; RSV-A: Respiratory syncytial virus-A; RSV-B: Respiratory syncytial virus-B; PIV-3: Parainfluenza virus 3; Flu-A/Pdm-09: Influenza A H1N1 strain; Hmpv: Human metapneumovirus; COVID-19: Novel coronavirus.

List of viruses	No. of cases with viral infection (N = 56)	% of patients with the isolated virus
Rhinovirus	17	30.35
Flu-A (H3N2)	14	25.0
RSV-B	6	10.71
Flu-B	4	7.14
PIV-3	6	10.71
Flu-A/PDM 09	4	7.14
HMPV	3	5.35
Adenovirus	2	3.57
RSV-A	2	3.57
COVID-19	4	7.14

A total of 63 bacteria were isolated in which gram-negative bacilli were most common, which include *Acinetobacter baumannii*, *Klebsiella pneumoniae*, and *Pseudomonas aeruginosa*. Among the gram positives, *Staphylococcus aureus *was the most common.

Rhinovirus was most commonly associated with bacterial coinfection in four cases (2.03%) followed by Flu-A and COVID-19. *Acinetobacter baumannii* was associated with a viral infection in most cases (five cases; 2.53%). This was followed by the detection of *Pseudomonas aeruginosa* and *Klebsiella pneumoniae* in two cases each (Table 5).

**Table 5:** Distribution of patients according to the type of bacteria isolated Note: Total may exceed the total number of patients affected with bacterial infection because of the isolation of multiple bacteria.

List of bacteria	No. of cases with bacterial infection (N = 63)	% of total bacteria isolated
Acinetobacter baumannii	14	22.22
Klebsiella pneumoniae	14	22.22
Pseudomonas aeruginosa	12	19.05
Staphylococcus aureus	5	7.94
Escherichia coli	8	12.70
*Enterobacter cloacae* complex	5	7.94
Serratia marcescens	2	3.17
Enterococcus faecium	1	1.59
Streptococcus pneumoniae	1	1.59
Staphylococcus haemolyticus	1	1.59
Sphingomonas paucimobilis	1	1.59

Breathlessness and cough were the most frequent complaints at the time of presentation. In cases with isolated viral exacerbation, 38 out of 39 cases (97.4%) had a shortness of breath, while 34 out of 39 (87.2%) cases had a cough. Fever was present in 14 out of 39 (32%) cases. However, sore throat was reported only in patients with isolated viral exacerbation, and chest pain was reported in patients with isolated bacterial exacerbations. Hypertension was the most common comorbidity reported in both bacterial and viral infections. Diabetes mellitus was mostly seen in patients who had a coinfection (Table 6).

**Table 6:** Clinical features and comorbidities in different types of infections

Clinical feature	Type of infection		
	Isolated viral	Isolated bacterial	Coinfection
Fever	14	19	6
Cough	34	36	13
Expectoration	9	10	4
Breathlessness	38	43	17
Chest pain	0	2	0
Sore throat	9	0	0
Altered sensorium	2	0	0
Comorbidities			
Hypertension	11	16	4
Diabetes mellitus	5	5	6
Parkinson’s disease	0	2	0
Coronary artery disease	0	4	0
Cerebrovascular accident	1	2	0
Chronic kidney disease	1	1	0
Cushing syndrome	1	0	0
Chronic liver disease	1	0	0
Carcinoma larynx	0	1	0
Alzheimer’s disease	0	1	0
Congenital heart disease	0	0	1

Among the 102 patients with infective exacerbations, patients with viral exacerbation had relatively lower mean TLC, while patients with exacerbation due to coinfection had the highest mean TLC. However, the results were not significant (p = 0.641). Among the patients with infective exacerbations, those with viral exacerbation had the least mean duration of hospital stay (7.33 ± 4.8 days), while patients with bacterial exacerbation spent the highest number of days in the hospital (10.082 ± 5.89 days). The 17 patients with coinfection had a mean duration of hospitalization of 6.8 ± 5.03 days. The results were not statistically significant (p = 0.071). Ten (26%) patients with viral exacerbation, 24 (52%) with bacterial exacerbation, and nine (53%) patients with a coinfection required respiratory support and hence needed admission to ICU. Severity was most commonly noticed in coinfection cases (p = 0.020). Two deaths were reported in viral infections, four in bacterial exacerbation, and three in coinfections (Table 7).

**Table 7:** Relationship between the infective exacerbations with clinical parameters and outcome P-value < 0.05 indicates statistically significant. S: Significant; NS: Non-significant.

Parameters	Mean Value			P-value
	Isolated viral infection (n = 39)	Isolated bacterial infection (n = 46)	Coinfection (n = 17)	
Mean age (years ± SD)	68.36 ± 3.45	71.8 ± 11.73	73 ± 8.33	0.084^NS^
Total leukocyte count (cells/mm^3^)	11.139 ± 4.8	12.49 ± 5.435	12.66 ± 7.3	0.641^NS^
Mean duration of hospital stay (in days)	7.33 ± 4.8	10.052 ± 5.89	6.8 ± 5.03	0.071^NS^
Type of cases				
Mild	12 (31%)	0 (0%)	0 (0%)	0.041^S^
Moderate	17 (43%)	22 (48%)	8 (47%)	0.062^NS^
Severe	10 (26%)	24 (52%)	9 (53%)	0.020^S^
No. of deaths among the severe cases	2	4	3	NA

The number of patients who had a severe disease was 43 (Table 7). Out of them, nine died. The rest 34 cases were advised pulmonary rehabilitation, oxygen therapy, inhaler-based medication as self-management home-based delivery, and were on telehealth monitoring. Five cases were lost to follow-up. In the rest 29 cases, information was documented after follow-up for one year that consisted of six viral infection, 17 bacterial infection, and six coinfection cases (Table 8).

**Table 8:** Outcome in one-year follow-up of severe cases (N = 29) P-value < 0.05 indicates statistically significant. S: Significant.

Condition of the patients after one year of follow-up	Viral infection (6 cases)	Bacterial infections (17 cases)	Coinfections (6 cases)	P-value
Clinically stable	6 (100%)	16 (94%)	2 (33%)	0.034^s^
Exacerbation (admission)	0	1 (6%)	4 (67%)	

All viral infection cases were clinically stable and did not require admission. Out of 17 bacterial infection cases, 16 (94%) were clinically stable and only one (6%) required hospital admission due to exacerbation. But in the six coinfection cases, two (33%) were clinically stable and the rest four (67%) cases required hospital admission, and the data was found to be statistically significant (p = 0.034). This shows most of the coinfection cases required rehospitalization during the period of follow-up (Table 8).

## Discussion

Acute exacerbation of COPD results in deterioration of pulmonary function, morbidity, and death. In our study, the mean age of the patients was 69.24 ± 11.08 years with a majority of the patients belonging to the age group of 61-80 years (Table 1). In a recent study conducted at the All India Institute of Medical Sciences (AIIMS), Bhubaneswar, the mean age was 65.49 ± 10.40 years [7]. As per another Indian study by Mood et al., the mean age of patients was 66.8 ± 11.4 years and the maximum prevalence was observed in the age group 70-79 years [8]. In another study that involved both European and American subjects, the proportion of females was 36.7% among Europeans and 33.3% among Americans, which is in accordance with our findings [9]. A study by Hajare et al. reported a male-to-female ratio of 2.3:1 [10]. The preponderance of males being affected can be attributed to the fact that males are more involved in outdoor activities and hence are more exposed to environmental pollutants [8]. Smoking is a risk factor for COPD and also its exacerbation as it decreases mucociliary clearance, which is amply proved in our study where smoking as a risk factor was noticed among 64% of patients [11]. In our study, the two main occupations that had increased the prevalence of COPD were farmers and housewives (Table 2). In a study published in 2016, occupations that were at COPD risk were seafarers, coalmine operatives, and cleaners [12]. In a study in Bangladesh, occupational exposures in farmers, hazardous exposures in tanners, and cotton dust exposures in garments were among the most prominent risk factors for the development of COPD [13]. In our study, the urban population comprised the majority (57.8%, Table 2), which correlates well with a study done in India where the prevalence of COPD was more in the urban areas. But there has been a significant increase in the prevalence in rural areas where it was reported to be 8.8% in a study done in India, whereas in our study, the prevalence is around 22% [14]. The disparity in the urban-rural divide is reversed in the United States, where the prevalence of COPD in rural communities is nearly double that in urban areas [15].

The complex interactions between environment, host, and microbes are responsible for exacerbations in COPD and increased morbidity and mortality [16]. As per studies, the major cause of acute exacerbations is infections [7]. In our study, infection was detected in 51.7% of cases (Table 3). In an Indian study, around 78.3% of cases had a respiratory infection [7]. Our study illustrates that only bacterial infection was found in 23.35% of cases; only viral etiology was found in 19.79% of cases, and bacterial and viral coinfection was found in 8.62% of cases. Other studies have reported bacterial infection in around 42%-49% of cases, viral infections in around 20%-64% of cases, and bacterial-viral coinfection in 27% of cases [7,17,18]. There has been an increased report of respiratory viruses as a causative agent in the acute exacerbation of COPD. With the application of molecular techniques in patients’ samples, viruses have been implicated in around 47%-66% of cases [11]. A total of 56 viruses were isolated (Tables 3, 4). The most common viruses isolated were rhinovirus, followed by Flu-A and RSV-B. Human rhinovirus (HRV) has been reported as a common viral isolate in various studies [18]. The study by Koul et al. also reported rhinovirus and influenza virus as the most common virus causing acute exacerbation of COPD [19]. The high rate of isolation of influenza virus may be attributed to the transmission of the influenza virus in the community and the need to have immunization [20]. In our study, more than one virus was isolated in three cases. Similar results have been found in a recent study in India [7]. The most common bacterial isolates in our study are *Acinetobacter baumannii*, *Klebsiella pneumoniae*, and *Pseudomonas aeruginosa* making up around 21.9% (for both *Acinetobacter *and *Klebsiella*) and 18.8%, respectively. Among the gram-positive bacteria, *Staphylococcus aureus* (7.8%), *Enterococcus faecium* (1.6%), and *Streptococcus pneumoniae* (1.6%) were the most common isolates. In the study by Jahan et al., the most common bacteria isolated were *Pseudomonas aeruginosa* (28%), followed by *Acinetobacter baumannii* and *Klebsiella pneumoniae* in seven cases each (21%) [7]. In another study, the most common bacterial isolates were *P. aeruginosa* (30.7%) followed by *K. pneumoniae* (20.3%) and *S. pneumoniae* (8.6%) [8].

It is to be noted that most of the studies implicate *Pseudomonas aeruginosa* as the most common bacteria causing exacerbation, whereas *Acinetobacter baumannii* and *Klebsiella pneumoniae *are the most common bacteria causing exacerbations as per (Table 5) of our study [21]. The predominance of *Acinetobacter *spp. in our study is a novel finding, and further studies are needed to know if this is the emerging trend in acute exacerbation of COPD as MDR (multidrug-resistant). *Acinetobacter baumannii* is implicated in the etiology of various other infections [22]. Jahan et al. reported coinfection with virus and bacteria in 24.9% of cases of acute exacerbations of COPD [7]. In our study, coinfection was detected in 9.63% of cases (Table 3). However, this may not represent a natural course as many patients are chronically infected with multiple pathogenic bacteria before a viral pathogen is detected. Conversely, viruses have been shown to be frequently followed by secondary bacterial infection. Most of the coinfections were seen to be associated with rhinovirus and influenza A virus, whereas it was mostly associated with both influenza A and influenza B in another study by Jahan et al. [7]. In another study, the viruses implicated alone or as coinfections are picornaviruses (especially rhinovirus), influenza virus, and respiratory syncytial virus [23]. Comorbidities were associated with eight cases of viral exacerbation with hypertension being the most common (Table 6). Similar findings were also reported by Koul et al. where hypertension was seen in 60.52% of cases followed by heart ailments (14.16%) [19]. No significant correlation was observed between the various subgroups. Breathlessness and cough were the most common clinical presentation in cases of exacerbation in our study. Sore throat, however, was reported only in viral exacerbation and not in bacterial or coinfection (Table 6). The outcome of viral exacerbation has improved over time, owing to an increase in adult vaccination and early treatment. Among the etiological agents, in our study, we noticed poor outcomes among the coinfection group probably as a consequence of systemic inflammation (Table 7). As per a study in Japan, gram-negative bacilli were significantly associated with prolonged hospitalization [24].

The severe category of patients who were discharged was put on telemedicine advice on pulmonary physiotherapy, medications, and home oxygen. Among them, the coinfection group had exacerbation that needed admission, and the rest of the cases were clinically stable (Table 8). There are not many studies that correlate the long-term outcome of acute exacerbation of COPD with infective causes. As per a review by Wang et al., it is observed that in cases where there is coinfection with bacteria and virus, the lung function impairment is greater and the duration of hospitalization is also longer [25]. In another study published in *Lung India*, where the outcomes were followed up for readmission for two years, 12% mortality was observed; readmission was seen in 54% of cases, and two or more readmissions were seen in 45% of cases [26].

Thus, a proportion of patients appear to be more susceptible to exacerbation. Hence, prevention and mitigation should be the key goals. The application of technological advancement in communication during the COVID pandemic enabled us to overcome the challenge through tailored prescription and telemedicine intervention.

## Conclusions

The clinical course of COPD is punctuated by exacerbation. These events are associated with accelerated loss of lung function, poor quality of life, increased health care costs, and mortality. Infection is the most important cause of exacerbation. *Klebsiella pneumoniae* and *Acinetobacter baumannii* among the bacterial isolates and rhino and influenza A viruses among the viral isolates were predominantly detected. During the telehealth follow-up, it was observed that those patients who had co-infections were more prone to readmission, whereas those who had isolated bacterial or viral etiology had better clinical stability. Pulmonary physiotherapy and appropriate medical measures for the mitigation of exacerbation can prevent further decline of disease progression.
